# Germinal centre frequency is decreased in pancreatic lymph nodes from individuals with recent-onset type 1 diabetes

**DOI:** 10.1007/s00125-017-4221-7

**Published:** 2017-02-17

**Authors:** Abby Willcox, Sarah J. Richardson, Lucy S. K. Walker, Sally C. Kent, Noel G. Morgan, Kathleen M. Gillespie

**Affiliations:** 1 Diabetes and Metabolism, Level 2 Learning and Research, University of Bristol, Southmead Hospital, Bristol, BS10 5NB UK; 2 Institute of Biomedical and Clinical Science, University of Exeter Medical School, Exeter, UK; 3 Institute of Immunity and Transplantation, UCL Division of Infection and Immunity, Royal Free Campus, London, UK; 4 Division of Diabetes, Department of Medicine, Diabetes Center of Excellence, University of Massachusetts Medical School, Worcester, MA USA

**Keywords:** Histology, Islet autoimmunity, Pancreatic lymph nodes, Type 1 diabetes

## Abstract

**Aims/hypothesis:**

Pancreatic lymph nodes (PLNs) are critical sites for the initial interaction between islet autoantigens and autoreactive lymphocytes, but the histology of PLNs in tissue from individuals with type 1 diabetes has not been analysed in detail. The aim of this study was to examine PLN tissue sections from healthy donors compared with those at risk of, or with recent-onset and longer-duration type 1 diabetes.

**Methods:**

Immunofluorescence staining was used to examine PLN sections from the following donor groups: non-diabetic (*n*=15), non-diabetic islet autoantibody-positive (*n*=5), recent-onset (≤1.5 years duration) type 1 diabetes (*n*=13), and longer-duration type 1 diabetes (*n*=15). Staining for CD3, CD20 and Ki67 was used to detect primary and secondary (germinal centre-containing) follicles and CD21 and CD35 to detect follicular dendritic cell networks.

**Results:**

The frequency of secondary follicles was lower in the recent-onset type 1 diabetes group compared with the non-diabetic control group. The presence of insulitis (as evidence of ongoing beta cell destruction) and diagnosis of type 1 diabetes at a younger age, however, did not appear to be associated with a lower frequency of secondary follicles. A higher proportion of primary B cell follicles were observed to lack follicular dendritic cell networks in the recent-onset type 1 diabetes group.

**Conclusions/interpretation:**

Histological analysis of rare PLNs from individuals with type 1 diabetes suggests a previously unrecognised phenotype comprising decreased primary B cell follicle frequency and fewer follicular dendritic cell networks in recent-onset type 1 diabetes.

## Introduction

Type 1 diabetes develops as a result of the immune-mediated destruction of insulin-secreting beta cells in the islets of Langerhans of the pancreas. Although the aetiology of the disease is not fully understood, it is underpinned by a combination of well-established genetic factors and as-yet unidentified environmental factor(s) [1–3].

Evidence of insulitis has been reported in histological pancreas specimens from donors with recent-onset type 1 diabetes, along with a marked reduction in islets that stain positively for insulin compared with specimens from healthy controls [4–6]. The immune infiltrate consists mainly of CD8^+^ cytotoxic T cells, CD20^+^ B lymphocytes and, to a lesser extent, CD4^+^ T cells, monocytes and natural killer cells [7]. Islet beta cells are the target of immune-mediated attack but it is within secondary lymphoid organs that lymphocytes are primed with their cognate antigen to initiate humoral and T cell responses [8, 9].

In humans, several pancreatic lymph nodes (PLNs; the number differs among individuals but can be >50) are located at the periphery of the pancreas [10]. Lymph nodes are highly organised structures that are densely populated with supportive stromal cells, antigen-presenting dendritic cells, macrophages and migrating lymphocytes. The lymph node environment continually evolves in response to foreign pathogens and, in the case of autoimmunity, self-antigens. Human lymph nodes consist of an outer capsule; a cortex of B cell follicles (outer zone) and T cells (inner zone); the medulla, through which medullary sinuses branch, facilitating the migration of cells; and the hilum, through which the lymph fluid exits the lymph node to flow through lymphatic vessels that connect each of the lymph nodes.

Within lymph nodes, B cell follicles exist in two major stages: primary and secondary. Primary follicles are populated by naive, mature follicular B cells and marginal zone B cells. Secondary B cell follicles are defined by the presence of germinal centres, which form when B cells encounter their cognate antigen. Upon antigen recognition, B cells migrate to the outer T cell zone, where they undergo cognate interactions with T cells before initiating the formation of germinal centres. Then, they rapidly proliferate and undergo class-switching, somatic hypermutation and affinity maturation to generate B cell clones of the highest affinity and specificity for their cognate antigen [11, 12]. Follicular helper T cells participate in B cell selection within the germinal centre light zone [13]. Germinal centres are important for the generation of memory B cells and long-lived antibody-producing plasma cells. Follicular B cells and specialised stromal cells, known as follicular dendritic cells (FDCs), are co-dependent. A positive feedback loop exists in which FDCs secrete the chemokine, CXCL13, which recruits CXCR5-expressing B cells, causing them to aggregate into follicular structures. CXCL13 stimulates the secretion from follicular B cells of tumour necrosis factor (TNF) and lymphotoxin (LT), which are essential for FDC development and maintenance [14–16].

Most reported studies of PLNs in type 1 diabetes disrupt the PLN structure to isolate the resident cells [17–19]. Histological studies of PLNs from human donors with type 1 diabetes are therefore rare. In particular, there are practically no studies of the PLN during the very early stages of disease when insulitis and residual beta cells are prevalent because of the rarity of human PLN tissues for research purposes. The aim of this study was to gain new insights into the immunological and architectural components of human PLNs, both preceding and during recent-onset and longer-duration type 1 diabetes.

## Methods

### Pancreatic lymph node tissues

**Fig. 1:**
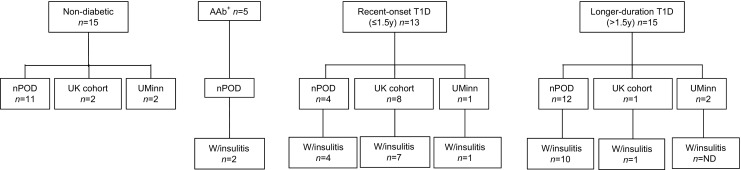
Flow diagrams detailing the number of donors with and without insulitis from the three cohorts utilised for the study. T1D, type 1 diabetes; W/insulitis, presence of insulitis

Human PLN tissue sections, representing all currently available resources, were obtained from the National Health Service Research Scotland Greater Glasgow and Clyde Biorepository (hereafter referred to as the UK cohort), the JDRF Network for Pancreatic Organ Donors with Diabetes (nPOD) programme (the nPOD cohort) and the University of Minnesota (the UMinn cohort), with kind permission from B. Hering, Division of Diabetes, Endocrinology and Metabolism, University of Minnesota, Minneapolis, USA. UK cohort specimens were archival, formalin-fixed, paraffin-embedded (FFPE) tissues, details of which have been published previously [4, 5, 20–22]. This study used nPOD-assigned nomenclature for each donor obtained from the nPOD cohort. PLNs from the nPOD cohort were provided as either snap-frozen or FFPE sections, depending on availability. PLN specimens from the UMinn cohort were provided as snap-frozen sections. Details of all donors are listed in Table 1 and Fig. 1. Tissues from each source were provided as 4–8 μm sections mounted on glass microscopy slides. To increase the statistical power, PLN samples from each cohort were analysed together. The demographics of the groups were: non-diabetic control donors (*n* = 15; six men, eight women), age range 2–31 years (mean ± SD 16.3 ± 2.7 years); autoantibody-positive donors (*n* = 5; three men, two women), age range 22–69 years (mean ± SD 38.3 ± 8.5 years); recent-onset type 1 diabetic donors (*n* = 13; six men, seven women), age range 5–27 years (mean ± SD 16.2 ± 2.0 years), duration of type 1 diabetes 0.02–1.50 years; and longer-duration type 1 diabetic donors (*n* = 15; nine men, six women), age range 11–34 years (mean ± SD 21.4 ± 1.7 years), duration of type 1 diabetes 4–19 years (mean ± SD 7.8 ± 1.2 years, median 6.0 years). Islet autoantibody data (micro-insulin autoantibody [mIAA], IA-2A, GADA, ZnT8A) were available for nPOD participants only. These data are included in Table 1 but were not analysed because most samples were tested years after diagnosis. In addition, autoantibodies to insulin cannot be discriminated from antibodies to therapeutic insulin. This study was carried out with ethical approval from NRES Midlands-Edgbaston Research Ethics Committee (13/WM/0300).

**Table 1:** Details of donors of PLN tissue included in the study

Donor code	Cohort	Participant type	Age (years)	Sex (M/F)	T1D duration (years)	Autoantibodies	Insulitis in pancreas (Y/N)
FD1	UK	RO T1D	12	F	0.2	ND	Y
FD2	UK	RO T1D	11	F	Recent	ND	Y
FD3	UK	RO T1D	15	M	0.5	ND	Y
FD4	UK	RO T1D	5	M	Recent	ND	Y
FD5	UK	RO T1D	14	F	Recent	ND	Y
FD6	UK	RO T1D	22	F	Recent	ND	N
FD7	UK	RO T1D	7	F	<1 week	ND	Y
FD8	UK	RO T1D	27	F	Recent	ND	Y
FD9	UK	LD T1D	20	M	6	ND	N
FC1	UK	Control	2	F	N/A	ND	N
FC2	UK	Control	18	F	N/A	ND	N
6052	nPOD	RO T1D	12	M	1	IA-2A, mIAA	Y
6070	nPOD	LD T1D	25.8	F	7	IA-2A, mIAA	Y
6076	nPOD	LD T1D	24	M	14	GADA, mIAA	Y
6087	nPOD	LD T1D	17.5	M	4	mIAA, ZnT8A	Y
6088	nPOD	LD T1D	31.2	M	5	GADA, IA-2A, mIAA	Y
6121	nPOD	LD T1D	33.9	F	4	Negative	N
6195	nPOD	LD T1D	19.2	M	5	GADA, IA-2A, mIAA, ZnT8A	Y
6211	nPOD	LD T1D	24	F	4	GADA, IA-2A, mIAA	N
6212	nPOD	LD T1D	20	M	5	mIAA	Y
6224	nPOD	RO T1D	21	F	1.5	Negative	Y
6228	nPOD	RO T1D	13	M	0	GADA, IA-2A, ZnT8A	Y
6243	nPOD	LD T1D	13	M	5	mIAA	Y
6245	nPOD	LD T1D	22	M	7	GADA, IA-2A	Y
6247	nPOD	RO T1D	24	M	0.6	mIAA	Y
6264	nPOD	LD T1D	12	F	9	Negative	Y
6265	nPOD	LD T1D	11	M	8	GADA mIAA	Y
6080	nPOD	AutoAb^+^	69.2	F	N/A	GADA, mIAA	N
6158	nPOD	AutoAb^+^	40.3	M	N/A	GADA, mIAA	N
6167	nPOD	AutoAb^+^	37	M	N/A	IA-2A, ZnT8A	N
6197	nPOD	AutoAb^+^	22	M	N/A	GADA, IA-2A	Y
6267	nPOD	AutoAb^+^	23	F	N/A	GADA, IA-2A	Y
6024	nPOD	Control	21	M	N/A	Negative	N
6094	nPOD	Control	2.9	M	N/A	Negative	N
6098	nPOD	Control	17.8	M	N/A	Negative	N
6137	nPOD	Control	8.9	F	N/A	Negative	N
6179	nPOD	Control	21.8	F	N/A	Negative	N
6182	nPOD	Control	2	M	N/A	Negative	N
6229	nPOD	Control	31	F	N/A	Negative	N
6233	nPOD	Control	14	M	N/A	Negative	N
6235	nPOD	Control	31	M	N/A	Negative	N
6234	nPOD	Control	20	F	N/A	Negative	N
6238	nPOD	Control	20	M	N/A	Negative	N
MD1	UMinn	LD T1D	23	F	19	ND	ND
MD2	UMinn	LD T1D	24	F	15	ND	ND
MD3	UMinn	RO T1D	27	M	1.5	ND	Y
MC1	UMinn	Control	ND	F	N/A	ND	ND
MC2	UMinn	Control	ND	F	N/A	ND	ND

AutoAb^+^, autoantibody-positive, non-diabetic; F, female; M, male; mIAA, micro-insulin autoantibody; N, no; N/A, not applicable; ND, not determined; Neg, negative; LD T1D, longer-duration type 1 diabetes; RO T1D, recent-onset type 1 diabetes; Y, yes

### Dual antibody immunofluorescence staining

To prepare FFPE tissues for labelling, sections were de-waxed with xylene and rehydrated through a decreasing ethanol series into distilled H_2_O. Heat-induced epitope retrieval was used to unmask antibody-binding epitopes as follows: sections were enclosed in a microwavable container containing 10 mmol/l Tris and 1 mmol/l EDTA (pH 9.0), and heated on full power (800 W) for 20 min, followed by 20 min cooling at room temperature. To prepare fresh-frozen tissues for labelling, sections were air-dried and immediately incubated with ice cold 10% formalin for 15 min, followed by three 5 min PBS (pH 7.4) washes. At this stage, frozen and FFPE sections were processed identically. Non-specific binding sites were blocked with 0.1% BSA in PBS supplemented with 5% normal goat serum for 30 min prior to incubation with primary antibody. Primary antibody binding was detected with fluorochrome-conjugated secondary antibodies specific to IgG of the primary antibody host species. Sections were mounted under glass coverslips with ProLong Gold Antifade mounting medium containing DAPI (Life Technologies). Details of all primary and secondary antibodies are shown in Table 2.

**Table 2:** List of antibodies used in the study

Antigen	Species	Clone	Conjugate	Manufacturer	Dilution
CD3	Rabbit	Polyclonal	Unconjugated	Dako	1/100
CD20	Mouse	L26	Unconjugated	Dako	1/200
Ki67	Rabbit	SP6	Unconjugated	Abcam	1/100
CD21	Mouse	2G9	Unconjugated	AbD Serotec	1/50
CD35	Rabbit	EPR6602	Unconjugated	Abcam	1/400
Rabbit IgG (H&L)	Goat	–	HRP	Life Technologies	1/200
Rabbit IgG (H&L)	Goat	–	DyLight 405	Vector Laboratories	1/100
Mouse IgG (H&L)	Goat	–	Alexa Fluor 488	Life Technologies	1/200
Rabbit IgG (H&L)	Goat	–	Texas Red	Vector Laboratories	1/100

H&L, heavy and light chains; HRP, horseradish peroxidase

### Triple antibody immunofluorescence staining

Use of a triple immunostaining system enabled two primary antibodies raised in the same species to label the same section without cross-reactivity [23, 24]. In this system, sections were processed as described above until the blocking stage and then incubated with rabbit anti-CD3 primary antibody. A goat anti-rabbit Alexa Fluor 594 TSA (‘tyramide signal amplification’) kit (ThermoFisher Scientific, Waltham, MA, USA) following the manufacturer’s instructions was used to detect and amplify the signal. After stringent PBS washes, sections were incubated with a cocktail of mouse anti-CD20 and rabbit anti-Ki67 primary antibodies, which were detected with goat anti-mouse Alexa Fluor 488 (ThermoFisher Scientific) and goat anti-rabbit DyLight 405 (ThermoFisher Scientific) secondary antibodies, respectively. Sections were mounted with ProLong Gold Antifade mounting medium without DAPI.

### Image capture and analysis

The area of each PLN section was measured using a Leica AF 6000 microscope fitted with a Leica DFC365FX 8/12 bit monochrome camera in combination with LASX software (Leica Microsystems, Milton Keynes, UK). An automated stage was used in combination with the software’s automatic tiling function to capture complete images of the whole sections. B cell follicles and germinal centres were observed and quantified based on detection of ≥3 CD20^+^ Ki67^+^ cells within a B cell follicle and characteristic histological features. The total number of B cell follicles/mm^2^ of PLN and the percentage of follicles that had formed germinal centres (secondary follicles) were quantified. T cell and B cell zones were distinguished by the presence of CD3^+^ and CD20^+^ cells, respectively. FDCs were identified as CD21^+^ CD35^+^ cells. Each B cell follicle was designated as either positive or negative for an FDC network following examination of adjacent sections.

### Statistical analysis

A Kruskal–Wallis H nonparametric test of independent samples was used to compare the number of follicles/mm^2^, the percentage of follicles with germinal centres and the percentage of follicles positive for FDCs among donor groups using IBM SPSS Statistics software (version 21, Chicago, IL, USA). A *p* value of <0.05 was considered statistically significant. The number of follicles with germinal centres was compared in PLNs from donors with recent-onset and longer-duration diabetes and controls. The insulitis score and age at onset were included as indicators of disease severity. Bonferroni correction was included to correct for multiple comparisons. Corrected (*pc*) and uncorrected (*p*) probability values are presented.

## Results

### Secondary follicles in PLNs from recent-onset type 1 diabetic donors

**Fig. 2:**
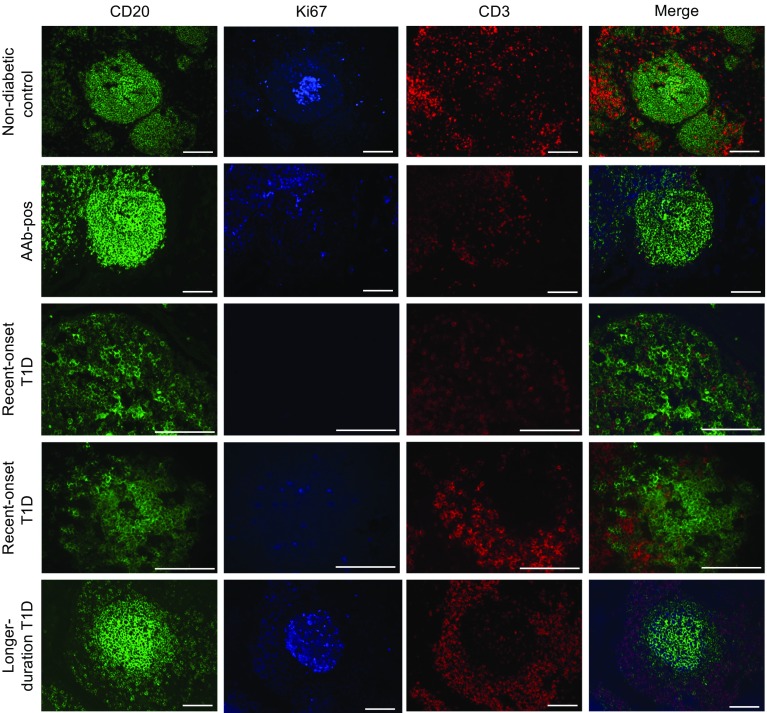
Representative examples of PLN B cell follicles images showing CD20^+^ follicles (green), Ki67 staining (blue), CD3^+^ T cells (red) and three-colour merged labelling from non-diabetic controls; autoantibody-positive (AAb-pos), recent-onset type 1 diabetes (T1D; primary follicle) donors; recent-onset T1D (secondary follicle) donors; and longer-duration T1D donors. Scale bars, 50 μm

Secondary B cell follicles, as indicated by germinal centre formation, were quantified in PLN tissue sections stained for CD3 (T cells), CD20 (B cells) and Ki67 (proliferating cells; Fig. 2). Control PLNs displayed a well-organised structure, with distinct differential B and T cell localisation and clearly defined follicles. The cellular organisation of PLNs from each recent-onset type 1 diabetic donor was altered compared with those from non-diabetic controls and longer-duration type 1 diabetic donors: primary follicles had less distinct, more diffuse structures, with poor differential localisation of the B cell and T cell subsets. This disorganised phenotype was also observed in islet autoantibody-positive PLNs.

For primary and secondary follicle analysis, PLNs were analysed by duration of diabetes (recent onset ≤1.5 years duration, longer duration >1.5 years duration). The total area of PLN tissue varied in non-diabetic controls (*n* = 15) and autoantibody-positive (*n* = 5), recent-onset type 1 diabetes (*n* = 13) and longer-duration type 1 diabetes (*n* = 15) donors (*p* = 0.005), but the mean frequency of total B cell follicles per mm^2^ of PLN did not differ among groups (*p* = 0.43; Table 3).

**Table 3:** Details of the total and mean area of PLN examined per donor group

Donor group	PLN area (mm^2^)	
	Total	Mean ± SEM
Non-diabetic control	192.6	12.84 ± 2.07
Autoantibody-positive	140.87	28.17 ± 9.64
Recent-onset T1D	70.1	5.39 ± 0.89
Longer-duration T1D	291.87	10.85 ± 3.8

T1D, type 1 diabetes

**Fig. 3:**
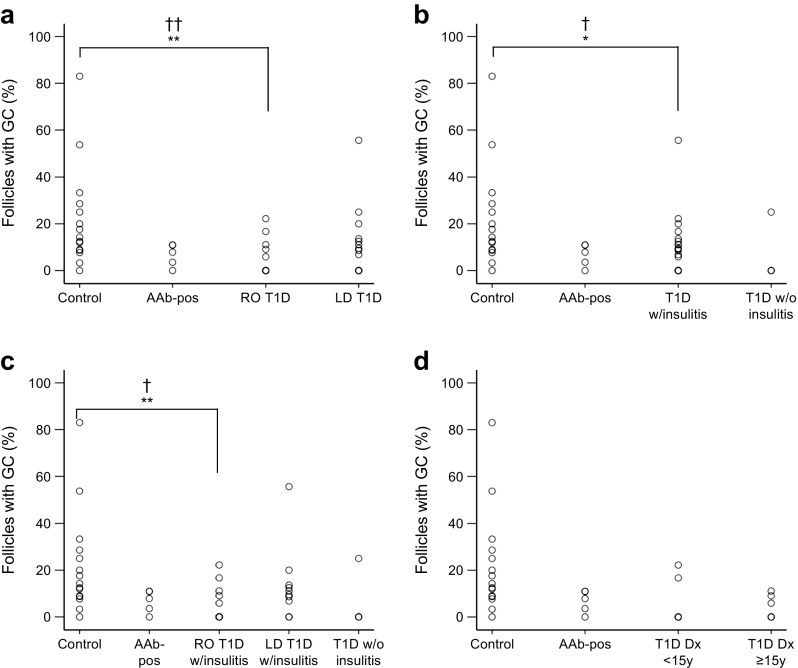
Plots demonstrating the percentage of total follicles in the secondary phase (per donor), as indicated by the presence of germinal centres (GCs), in donors with and without type 1 diabetes (T1D). (**a**) Donors stratified by duration of diabetes (recent-onset [RO] T1D *n* = 13, longer-duration [LD] T1D *n* = 15). (**b**) Donors stratified by the presence (w/insulitis) or absence (w/o insulitis) of pancreatic insulitis (T1D w/insulitis *n* = 22, T1D w/o insulitis *n* = 4). (**c**) Donors stratified by both T1D duration and the presence or absence of insulitis (RO T1D w/insulitis, *n* = 12; LD T1D w/insulitis, *n* = 10; T1D w/o insulitis, *n* = 4). (**d**) RO T1D donors diagnosed before age 15 years (<15 y, *n* = 7) and at age 15 years or older (≥15 y, *n* = 6). Control donors, *n* = 15, Autoantibody-positive (AAb-pos) donors, *n* = 5 for all plots. Each data point represents a single donor. Donors with the same value are overlaid, as indicated by a denser outline. *p < 0.05, **p < 0.01; ^†^ *pc* < 0.05, ^††^ *pc* < 0.01. Dx, diagnosis

Overall, the frequency of total B cell follicles containing a germinal centre was lower in PLNs from recent-onset type 1 diabetic donors compared with controls (5.0 ± 2.1% [median 0.0%] vs 21.9 ± 5.6% [median 14.3%], respectively, *p* = 0.003, *pc* = 0.009; Table 4 and Fig. 3a), while longer-duration type 1 diabetic donors were not different from controls (10.9 ± 3.8% [median 8.6%] *p* = 0.21). Germinal centre frequency was not significantly different in islet autoantibody-positive donors (6.7 ± 2.1% [median 7.8], *p* = 0.055, *pc* = 0.165) compared with controls.

**Table 4:** Frequency of total B cells follicles and the percentage containing germinal centres

Donor group	B cell follicle frequency, per mm^2^ (median)	Germinal centre frequency, % of total (median)
Non-diabetic control^a^	1.83 ± 0.22 (1.77)	21.91 ± 5.6 (14.3)
Autoantibody-positive	2.59 ± 0.42 (2.4)	6.66 ± 2.14 (7.84)
T1D		
RO	1.99 ± 0.36 (1.55)	4.99 ± 2.1 (0.0)
LD	1.87 ± 0.28 (1.64)	10.85 ± 3.8 (8.64)
T1D with insulitis		9.22 ± 2.68 (7.7)
RO	2.1 ± 0.37 (1.62)	5.4 ± 2.2 (0.0)
LD	1.95 ± 0.34 (1.72)	13.78 ± 5.02 (10.32)
RO T1D		
Dx <15 years	1.61 ± 0.39 (1.37)	5.56 ± 3.64 (0.0)
Dx ≥15 years	2.43 ± 0.63 (1.86)	4.35 ± 2.06 (2.94)

Data are means ± SEM

^a^Non-diabetic control group results remained consistent throughout the analysis and are therefore described only once

Dx, diagnosis; LD, longer duration; RO, recent onset; T1D, type 1 diabetes

### Secondary follicles in PLNs from type 1 diabetic donors with insulitis

Next, tissues were stratified by the presence or absence of insulitis in the pancreas, irrespective of disease duration. Data regarding evidence of insulitis was ascertained from previous studies involving the same donors, and from nPOD online pathology information [5, 22, 25]. The insulitis status of two of the type 1 diabetic donors could not be established (MD1, MD2) and were not therefore included in this part of the analysis. There was a reduction in the frequency of secondary follicles in PLNs from type 1 diabetic donors with insulitis (*n* = 22) compared with non-diabetic controls (9.2 ± 2.7% [median 7.7%] vs 21.9 ± 5.6% [median 14.3%], *p* = 0.014; *pc* = 0.042; Fig. 3b). Only four type 1 diabetic donors without insulitis were included; this was considered insufficient for analysis.

### Secondary follicles in PLNs from recent-onset donors with insulitis stratified for duration of diabetes

Type 1 diabetic donors were stratified by both duration of diabetes and evidence of insulitis; those with evidence of insulitis were subdivided into recent-onset (*n* = 12) and longer-duration (*n* = 10) groups. The mean frequency of total follicles (*n*/mm^2^) was not significantly different in the recent-onset type 1 diabetes with insulitis or longer-duration type 1 diabetes with insulitis groups compared with controls (2.1 ± 0.4/mm^2^ [median 1.62/mm^2^], 2.0 ± 0.3/mm^2^ [median 1.7/mm^2^] and 1.8 ± 0.2/mm^2^ [median 1.8/mm^2^], respectively; *p* = 0.8). A reduction in the mean frequency of secondary follicles was observed in recent-onset donors with insulitis compared with non-diabetic donors (*n* = 15; 5.4 ± 2.0% [median 0.0%] vs 21.9 ± 5.6% [median 14.3%], *p* = 0.005, *pc* = 0.015) but not in longer-duration donors with insulitis compared with non-diabetic donors (13.8 ± 5.0% [median 10.3%] vs 21.9 ± 5.6% [median 14.3%], *p* = 0.24; Fig. 3c).

### Secondary follicles in PLNs from type 1 diabetic donors diagnosed before the age of 15 years

In the longer-duration diabetes group, the time between diagnosis of type 1 diabetes and death varied from 4 to 19 years. Considering the observed difference in secondary follicle frequency in PLNs from donors with recent-onset and longer-duration diabetes, the phenotype of PLNs from the donors with longer-duration diabetes at the time of retrieval was unlikely to reflect the phenotype at diagnosis. Therefore, when examining the effect of age at diagnosis of diabetes on the frequency of secondary follicles, it was necessary to further analyse the recent-onset type 1 diabetic donors (*n* = 13; seven aged <15 years, six aged ≥15 years) in isolation because the length of time between diagnosis of type 1 diabetes and death was very short (≤1.5 years) and thus more likely to accurately reflect the phenotype at diagnosis. There was no difference in secondary follicle frequency between recent-onset type 1 diabetic donors diagnosed at age <15 years and recent-onset type 1 diabetic donors diagnosed at age ≥15 years (*p* = 0.84; Fig. 3d), suggesting that diagnosis at a younger age had no effect on the lower frequency of secondary follicles in PLNs from donors with recent-onset type 1 diabetes.

### A proportion of primary follicles lack FDC networks in donors with recent-onset type 1 diabetes

**Fig. 4:**
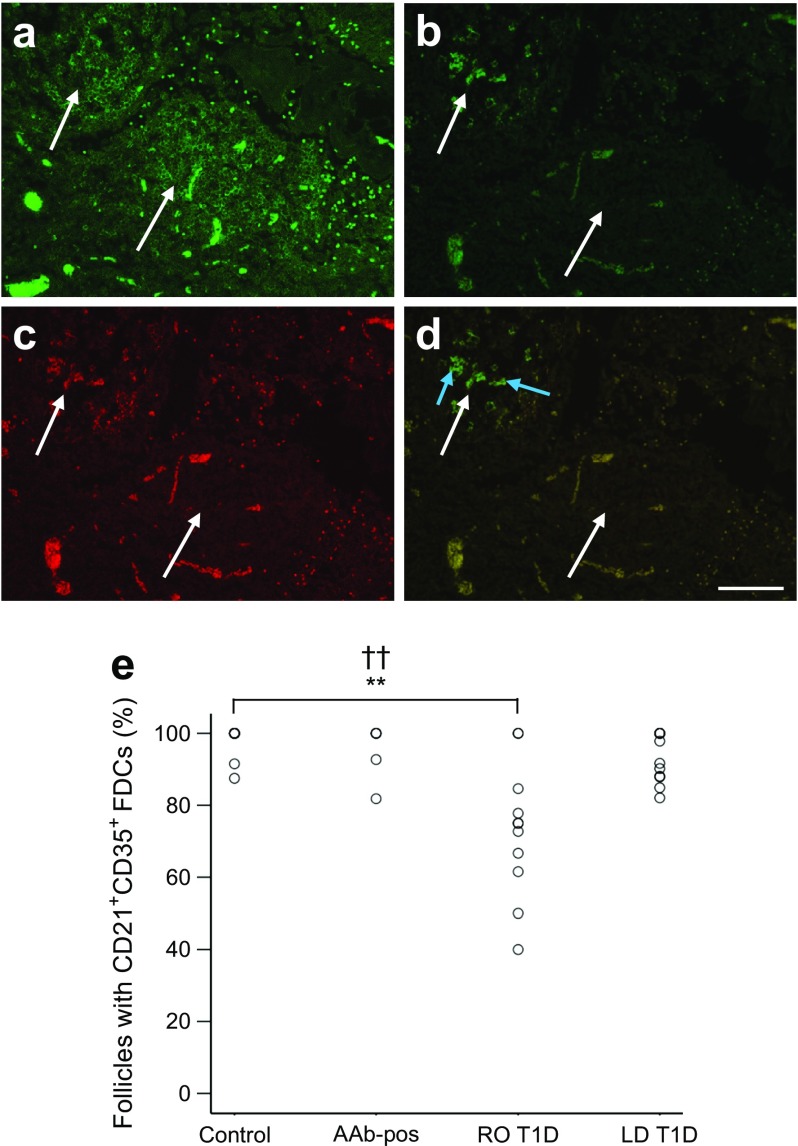
FDC networks in human PLNs. (**a**) Image shows two CD20^+^ primary B cell follicles (stained green, indicated by the white arrows in all images) from a recent-onset type 1 diabetic donor. (**b**–**d**) Images showing the same region in the section adjacent to the one shown in (**a**), stained for (**b**) CD21 (green) and (**c**) CD35 (red) and (**d**) merged. (**d**) The top left follicle contains CD21^+^ CD35^+^ FDCs (indicated by blue arrows), which were not detected in the bottom follicle. Scale bar, 50 μm. (**e**) The histogram shows the percentage of B cell follicles which contained CD21^+^ CD35^+^ FDC networks in each donor group (control, *n* = 15; Autoantibody-positive [Aab]-pos) donors, *n* = 5; recent-onset [RO] T1D donors, *n* = 11; longer duration [LD] T1D, *n* = 12). **p < 0.01; ^††^ *pc* < 0.01

PLN tissue sections adjacent to those labelled for T cell, B cell and proliferating cell markers (CD3, CD20 and Ki67, respectively) were stained for the complement receptors CR1 (also known as CD35) and CD2 (also known as CD21). Both markers are highly expressed by FDCs. Given their co-dependent relationship, FDC networks were employed as another marker of B cell follicles. Absence of FDC networks from a follicle was indicated by the lack of CD21^+^ CD35^+^ immunopositive cells in regions of the outer cortex that had CD20^+^ B cell follicles on an adjacent section (Fig. 4a–d). Donors from the UMinn cohort were not included in the FDC analysis because the sections were not always consecutive. In PLNs of the non-diabetic donor group, 98.1 ± 1.3% (median 100%; *n* = 11) of follicles showed evidence of an FDC network, compared with 94.9 ± 3.6% (median 100%; *n* = 5), 93.6 ± 2.0% (median 94.75%; *n* = 12) and 73.0 ± 5.6% (median 75%; *n* = 11; *p* = 0.001; *pc* = 0.003) of follicles in the autoantibody-positive, longer-duration type 1 diabetes and recent-onset type 1 diabetic donor groups, respectively (Fig. 4e). Each donor PLN containing one or more FDC-negative regions also contained one or more FDC-positive regions. This finding suggests that ineffective labelling of the CD21 and CD35 proteins on certain donor tissues was not responsible for the lack of detected FDCs within some follicles. Only subsets of primary follicles were found to be FDC-negative, and all secondary follicles contained CD21^+^ CD35^+^ cells.

## Discussion

The aim of this study was to examine the phenotype of PLNs from donors with varying duration of type 1 diabetes, including islet autoantibody-positive, non-diabetic donors considered to have a high risk of future type 1 diabetes. A previously unrecognised phenotype was revealed in PLNs from donors with recent-onset type 1 diabetes, which was not observed in PLNs from non-diabetic donors or those with a longer duration of diabetes. The recent-onset phenotype included a disorganised structure and a reduced frequency of secondary follicles, as well as a decreased frequency of follicles containing FDC networks. When the presence of insulitis was used instead of time from onset, a reduced secondary follicle frequency compared with controls was also observed. Diagnosis of type 1 diabetes at a younger age did not appear to be associated with lower germinal centre occurrence. In addition, the follicle frequency in PLNs did not appear to be affected by duration of diabetes or age at onset, suggesting that the lack of secondary follicles could not be attributed to an overall reduction in, or an inability to form, primary follicles.

Previous histological studies of PLNs from donors with type 1 diabetes are very limited. A recent study, however, conducted a thorough examination of extracellular matrix proteins in tissues from nPOD donors, and reported no difference in germinal centre frequency in PLN tissue between donors with and without type 1 diabetes [19]. Several differences in study design between this former study and the current study may explain the contradictory observations. In the former study, donors with diabetes duration of up to 9 years were grouped together as younger donors. In the present study, donors with diabetes duration of ≤1.5 years were designated the recent-onset diabetes group and all other type 1 diabetic donors were designated the longer-duration diabetes group. Comparison of the nPOD donors detailed in the former and current studies showed that only four type 1 diabetic and two control donors were utilised in both studies. The former study, however, analysed multiple lymphoid tissues from the same donors and did not identify which donors supplied the PLNs studied. The labelling methods also differed in that CD3, CD20 and Ki67 were co-stained in the present study, while sequential sections were singly labelled for CD3 and CD20, without the addition of a proliferation marker in the former study. As rapid proliferation is the hallmark of a functional germinal centre, the inclusion of Ki67 staining is a strength of the current study. Another strength is the use of human tissue samples from three cohorts of rare donors.

Limitations of the study include the cross-sectional nature of the investigation. This limitation cannot be addressed in human studies because access to PLNs is largely restricted to post-mortem retrieval. Sample size, particularly in stratified analyses, was also a limitation but all PLNs available for study were included. The labelling of only one PLN section per group of markers limited the quantity of data that could be generated from a single lymph node, but this could not be improved because only a few sections were available per donor. Another consideration was the use of both snap-frozen and FFPE tissue sections, but low sample numbers necessitated the use of both sample types. An in-house comparative analysis showed that germinal centre identification was possible in both snap-frozen and paraffin-embedded PLN tissue but, overall, preservation of the tissue architecture was better in FFPE sections (data not shown). Therefore, FFPE sections were selected where possible and snap-frozen tissues were chosen for staining when FFPE tissue was unavailable. As the tissue samples were from several different cohorts, consistent data on insulin-containing islets in the adjacent pancreas were not available. Insulitis scores may reflect insulin positivity but the pattern of insulitis is known to be heterogeneous. This study also lacks a functional analysis of PLN immune cells. However, such studies are being addressed by other groups [17, 18, 25], while the current study sought to fill a gap in the knowledge of in situ PLNs from relevant type 1 diabetic donors.

Research into the formation of primary and secondary follicles is complex, involving cells and signalling proteins, and therefore outside the scope of this observational study. Of particular importance are T follicular helper (Tfh) cells, a subset of CD4^+^, CXCR5^+^ T cells that are integral to B cell affinity maturation within germinal centres [26, 27]. T cells with a Tfh phenotype were recently reported to be over-represented in the blood of individuals with type 1 diabetes [28, 29]. Activated follicular B cells require intercellular CD40 (on B cells), CD40L (on Tfh cells) co-stimulation to promote their survival and affinity maturation [30, 31]. Although many studies have examined the mechanisms responsible for the formation and subsequent dissolution of germinal centres [32–34], none to date have reported abrogation in germinal centre formation in PLNs from mouse models of diabetes. *Ptpn22* knockout (KO) mice, however, showed increased germinal centre activity, which was attributed to increased proliferation and survival of CD4^+^ Tfh cells [35]. If *Ptpn22* KO and loss of lymphoid protein tyrosine phosphatase (LYP) function results in increased germinal centre activity, it could be argued that an increase in LYP function could contribute to decreased germinal centre activity. Another study showed that by disrupting the lymphotoxin pathway during NOD mouse development, formation of primary follicles and FDC networks in the spleen was diminished but primary and secondary follicles formed normally in the draining lymph nodes, including PLNs [36].

While acknowledging that this small observational study requires future replication and mechanistic investigation, we have considered possible explanations for the observation of fewer secondary follicles with fewer germinal centres and fewer FDC networks in PLNs from donors with recent-onset diabetes. It is possible that reduced antigen presentation to B cells or a breakdown in the normal process of secondary follicle stimulation, with maturing autoreactive B cells being pushed into antibody-producing plasma cells faster than usual, could contribute to depletion of secondary follicles. It is not, however, possible to address these possibilities histologically. A potential role for B cells in the pathogenesis of type 1 diabetes is supported by a recent anti-CD20 trial [37] and by the association of CD20^+^ B cell rich insulitis with rapid progression [38]. B cells may contribute to functional effects on antigen presentation, co-stimulation or local cytokine secretion and may therefore potentiate the autoreactive response in type 1 diabetes.

In conclusion, we have described a histological observation of diminished germinal centre activity close to diagnosis of type 1 diabetes.

## Abbreviations

FDC: Follicular dendritic cell
FFPE: Formalin-fixed, paraffin-embedded
GADA: GAD autoantibody
IA-2A: Islet antigen-2 autoantibody
LT: Lymphotoxin
nPOD: Network for Pancreatic Organ Donors with Diabetes
*pc*: Corrected *p* value
PLN: Pancreatic lymph node
Tfh: T follicular helper
TNF: Tumour necrosis factor
uMinn: University of Minnesota (cohort)
ZnT8A: Zinc transporter 8 autoantibody
